# Multi-Head Spatiotemporal Attention Graph Convolutional Network for Traffic Prediction

**DOI:** 10.3390/s23083836

**Published:** 2023-04-09

**Authors:** Ariyo Oluwasanmi, Muhammad Umar Aftab, Zhiguang Qin, Muhammad Shahzad Sarfraz, Yang Yu, Hafiz Tayyab Rauf

**Affiliations:** 1 School of Information and Software Engineering, University of Electronic Science and Technology of China, Chengdu 610054, China; ariyo@uestc.edu.cn (A.O.); qinzg@uestc.edu.cn (Z.Q.); 2 Department of Computer Science, National University of Computer and Emerging Sciences, Islamabad, Chiniot-Faisalabad Campus, Chiniot 35400, Pakistan; ms.umaraftab@yahoo.com (M.U.A.); shahzad.sarfraz@nu.edu.pk (M.S.S.); 3 Centre for Infrastructure Engineering and Safey, School of Civil and Environmental Engineering, The University of New South Wales, Sydney, NSW 2052, Australia; 4 Independent Researcher, Bradford BD8 0HS, UK; hafiztayyabrauf093@gmail.com

**Keywords:** traffic forecasting, graph convolutional network, gated recurrent unit, multi-head attention

## Abstract

Intelligent transportation systems (ITSs) have become an indispensable component of modern global technological development, as they play a massive role in the accurate statistical estimation of vehicles or individuals commuting to a particular transportation facility at a given time. This provides the perfect backdrop for designing and engineering an adequate infrastructural capacity for transportation analyses. However, traffic prediction remains a daunting task due to the non-Euclidean and complex distribution of road networks and the topological constraints of urbanized road networks. To solve this challenge, this paper presents a traffic forecasting model which combines a graph convolutional network, a gated recurrent unit, and a multi-head attention mechanism to simultaneously capture and incorporate the spatio-temporal dependence and dynamic variation in the topological sequence of traffic data effectively. By achieving 91.8% accuracy on the Los Angeles highway traffic (Los-loop) test data for 15-min traffic prediction and an R2 score of 85% on the Shenzhen City (SZ-taxi) test dataset for 15- and 30-min predictions, the proposed model demonstrated that it can learn the global spatial variation and the dynamic temporal sequence of traffic data over time. This has resulted in state-of-the-art traffic forecasting for the SZ-taxi and Los-loop datasets.

## 1. Introduction

**Figure 1:**
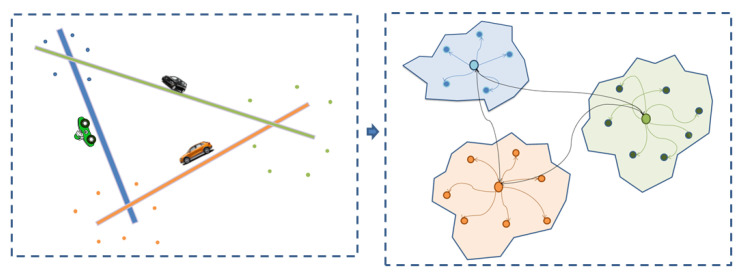
Sample road network mapped into graph connections.

In most developed cities, developing an intelligent transportation system (ITS) is of utmost importance to achieve sophisticated traffic control and extensive transportation management. This extends to forecasting the number of commuters and vehicles using a route, as displayed in Figure 1, and predicting the traffic congestion at a determined time. As a result, this ensures the security of passengers and the efficiency of travel routes by determining key factors based on an analysis of the road during specific periods [1,2].

Generally, traffic prediction involves studying various transportation variables such as vehicular speed flow, traffic patterns, region industrialization, and various other topographical features involved in the exploratory analysis and forecasting of traffic trends [3,4]. Subsequently, this drives precise real-time traffic forecasting, an integral part of intelligent traffic systems, as well as traffic control and management [5,6]. Correspondingly, most countries’ transportation ministries are currently deploying such intelligent transportation models to combat the various challenges of modern day transportation [7].

However, because of the topological design of most urban road networks, the complexity of road connectivity and the dynamic variations in traffic trends over time, traffic forecasting requires sophisticated modeling to achieve accurate prediction and analysis [8]. This is because traffic forecasting systems involve spatial and temporal components that are represented by non-Euclidean graphical structures that change from neighborhood to neighborhood in the time-space coordinate spectrum [9].

Therefore, the motivation of this work is to learn the complex topological spatial and temporal dependencies of traffic analyses. To achieve this, we implement a special type of graph neural network (GNN) called a graph convolutional network (GCN), particularly suitable for graphical structures. In GNNs, the structure of data is represented as nodes that occupy arbitrary positions in space, while the edges are a representation of the nodes’ connectivity and relationships [10].

A GCN is a unique extension of a CNN [11] that learns representation in non-Euclidean structures from neighboring nodes as embeddings containing all the information about the graph network while maintaining the weight-sharing filter operations of the vanilla convolutional neural network (CNN) [12,13]. Generally, graphs can describe several scenarios such as the connection of molecules; however, in our work, the graph network is represented as a transportation system network where the road represents nodes, and the interconnection of these roads is represented by the edges.

There are presently several models used for traffic forecasting which are based on different approaches, some of which focus on spatial feature extraction and analyses. Other models study the temporal dependence of the graphical road network, while both spatial and temporal dependence of the graphical road networks are considered in some [14]. This study proposes the multi-head spatiotemporal attention graph convolutional network (MHSTA–GCN) for traffic prediction to solve this problem. Our MHAST-GCN model incorporates a graph convolutional network (GCN), gated recurrent units (GRU), and multi-head attention (MHA) models to achieve high accuracy traffic estimation and prediction.

Maintaining the matrix multiplication operation of CNNs [15], the GCN helps to extract and learn complex features from inter-connected neighboring nodes in each iteration through the convolution filter weight update [16]. Furthermore, the gated recurrent units play a vital role in capturing and modeling the dynamic temporal features of the road network [17], while the attention mechanism causes the model to focus on the exact essential characteristics which correlate to the traffic flow at a particular time [18,19]. In a nutshell, the following contributions are made in this research:

1. This study proposes a spatiotemporal multi-head attention graph convolutional network to model the non-Euclidean temporal interrelationships between road networks to predict traffic flow.
2. The proposed model translates traffic features into learned embedding representations with a graph convolution network and then transforms them as time series sequences into a recurrent network using internal states and memory to model the temporal representations.
3. This study estimates the importance of features via a multi-head attention mechanism to generate a context vector of significant weights, emphasizing relevant features which play the most crucial role in the traffic forecast at a particular time.

For a graph-like structured task such as road networks and, by extension, traffic prediction, the GCN enables effective structural information extraction by leveraging an extensive neighborhood aggregation rule to obtain representative feature engineering and learning. The network features are learned by taking the node features and an adjacency matrix as inputs. Applying the tremendous expressive potential of GNNs, the feature representation of each node in the network is captured and the associated embeddings are learned. These embeddings are then processed as a sequence of inputs using a recurrent neural network with an internal state and memory to produce accurate traffic estimations.

The graph convolutional network is beneficially able to capture the spatial dependencies in traffic data by modeling the relationships between the various longitude–latitude locations on the road network. Similarly, the gated recurrent unit is efficient at capturing the temporal dependencies of traffic data through the time-based modeling of sequential patterns of traffic flow over time. Finally, the multi-head attention mechanism is used to capture the long-range dependencies in traffic data patterns by permitting the model to focus on the different but most important parts of the traffic input sequence.

By combining all of these three techniques, the proposed method aims to improve the accuracy of traffic prediction. While each of these techniques has been used individually in the past, the novelty of the proposed method lies in the way they are combined to improve traffic prediction. Therefore, the contribution of this article is the development of a novel method for traffic prediction that leverages multiple techniques to improve the accuracy of predictions.

The organization of the rest of this paper is structured as follows: Section 2 reviews the summary of essential traffic forecasting models, while Section 3 presents a comprehensive description of the proposed MHSA–GCN model. Section 4 highlights the model’s experimental implementation and an analysis is presented, and finally, Section 5 summarizes the paper in the conclusion.

## 2. Related Work

### 2.1. Traffic Prediction

Several existing traffic forecasting methods can be classified as either model-driven or data-driven based on how the road network’s nodes and edge relationships are mapped. This includes the use of classical machine learning models such as the K-nearest neighbor [20], support vector regressor [21], Bayesian models [22], and hybrid auto-regressive integrated moving average (H-ARIMA) [23] models. The downside of these techniques is that although they consider the temporal transformation of traffic data, the spatial dependencies are neglected. This problem also applies to artificial neural network multilayer perception models.

To accurately predict traffic data, the spatial characteristics are considered via the use of a convolutional neural network (CNN) to learn space invariant feature maps [24]. However, CNNs are most advantageous for Euclidean data types, particularly images, resulting in a less accurate traffic prediction. Recurrent models such as gated recurrent unit (GRU) [25], long short-term memory (LSTM) [26], and their variants employ extensive gated memory cells to learn compelling temporal features as a sequence, where previous time steps influence the current time step prediction [27]. Osipov et al. [28] applied a self-learning recurrent neural network with a spiral structure of layers to resolve some deficiencies of time-series samples. As such, the model permits continuous training and advanced associative recall of cues from previous cells to improve the forecast accuracy. Similarly, the gradient boosting, recurrent neural network (RNN), and multilayer perceptron neural network (MLP-NN) models have been implemented to achieve traffic flow predictions via the use of a sampled number of vehicles and region intersections as input features for training [29]. J. Zhang et al. explored the conversion of road networks into grid-like structures for effective convolutional sliding. They represented the grid as a region of longitudinal and latitude bisections and then extracted spatial features and their correlation with different grid regions [30].

### 2.2. Graph Convolutional Networks

Maximizing the capability of CNNs and RNNs, both architectures are often merged to take advantage of the temporal and spatial dependence of road networks for speed and traffic prediction [31]. For instance, the LC-RNN model of Zhongjian et al. [32] employed an embedded convolution technique to learn essential spatial features via topology-aware look-up operations. The learned spatial representation is then integrated into a recurrent network as a time-series sequence, resulting in extensive neighborhood referencing dynamics. Likewise, a hybrid nonvolutional neural network (CNN) and recurrent neural network (RNN) architecture was developed by Guo et al. [33] to model vehicle global positioning system (GPS) trajectory data. The technique proposed applying three-dimensional convolutional networks to model complex urban topography. While 3D CNNs are pretty effective, they require extensive computation which could be time-consuming. To tackle this problem, graph convolutional neural networks, which are more suitable for non-Euclidean data, are often promoted for analyzing traffic patterns [34].

A kernel-weighted graph network which learns convolutional kernels and their linear weights achieved satisfactory accuracy in capturing the non-grid traffic data [35]. Furthermore, to tackle complex, nonlinear traffic data, the DualGraph model explored the interrelationship of nodes and edges with two graph networks. The technique employed the Simulation of Urban Mobility (SUMO) software to obtain traffic data which were effectively evaluated [36]. The graph convolution recurrent architecture was optimized by Kan et al. [37] through a data-driven approach while training, affirming the latent relationship between road segments. Road segment importance was analyzed and weighted to capture global temporal features using attention models [38]. Additionally, external historical factors such as facilities and weather conditions were integrated into an attribute-augmented spatiotemporal network, resulting in a state-of-the-art model in traffic prediction [39].

### 2.3. Attention Mechanism

In a bid to improve neural networks’ capability to predict traffic forecasts accurately, attention models are often developed to create a vector of importance and illustrate how specific features influence traffic inference at a particular time [40,41]. For instance, attention models were utilized to analyze the significance of road segments and assign weights that capture previous timestamp characteristics [38], culminating in the simultaneous capturing of point significance and temporal dynamics. Using recent, daily periodic, and weekly periodic dependencies, the dynamic spatiotemporal correlations of data points were weighted with an attention mechanism on the Caltrans Performance Measurement System (PeMSD4 and PeMSD8) datasets [42].

Furthermore, normalized spatial attention weights were evaluated by Weiwei et al. to measure the influence of grids in sampled data and points of interest. It was concluded that grids with strong connections had a more considerable influence on other such grids [43]. For the graph attention convolutional network (GAC-Net), new learnable parameters were introduced with a self-attention network for spatial feature extraction, producing a valid result in the temporal correlation of the sequential series [44]. A self-attention mechanism was also incorporated into a graph convolutional network by Ke et al. [45], which improved the extraction of complex spatial correlations inside the traffic network. The self-attention-based spatiotemporal graph neural network (SAST–GNN) added channels and residual blocks to the temporal dimension to improve the short-term and mid-term prediction accuracies [46]. Inspired by Google’s transformer framework, Ling et al. designed a traffic transformer to capture the continuity and periodicity of traffic sequences as time series, demonstrating a significant ability to forecast traffic flow [47]. A comparative analysis of the proposed model with recent literature models is presented in Table 1 and Table 2 with well-known evaluation metrics.

## 3. Multi-Head Spatiotemporal Attention GCN

**Figure 2:**
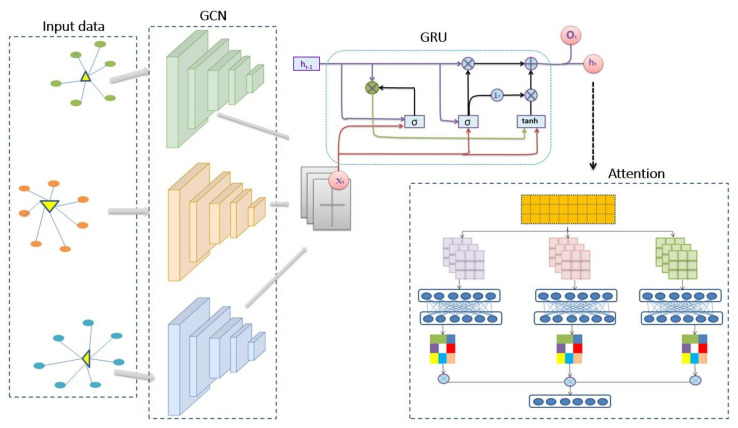
Proposed MHSA–GCN architectural overview of GCN, GTU, and attention.

This section describes the network design of the MHSAGCN model and its architectural representation, as shown in Figure 2.

### 3.1. Graph Convolutional Network (GCN)

With the interconnection of road networks represented as a graph, using a graph convolutional network, we represent the association of road networks as graph *G* presented as G=(V,E) , where each major road in the network is represented as a node, *V*, and the connections between the roads are referred to as edges, *E*. *V* is represented as the set of major roads $V=[v_{1},v_{2},v_{3},\cdots ,v_{N}]$ , where *N* is the total number of road nodes available in the graph.

For a standard GCN, two inputs are required: the feature matrix, *X*, and the adjacency matrix, *A*. The feature matrix, *X*, represents node attribute features and, in this work, refers to the traffic speed recorded for each node over time, expressed as $X\in R^{N*P}$ . *P* is the total traffic speed attribute recorded, while the adjacency matrix, *A*, is the symmetrical representation of the graph connection. In the road network, every connected nodes are valued at one, and zero at nodes without connection. 1 at connected nodes and 0 otherwise. The adjacency matrix is therefore expressed as $A\in R^{N*N}$ .

With the observed traffic speed historical time series *n*, the forecasting problem is defined as learning the traffic mapping function, *f*, of the next time steps, *T*, denoted in Equation (1) as:

(1) $$\left[X_{t+1},X_{t+2},\cdots ,X_{t+T}\right]=f\left(G(X_{t-n},\cdots ,X_{t-1},X_{t})\right)$$

Finally, given feature matrix *X* and adjacency matrix *A*, the GCN computes the first-order neighborhood weighted average and matrix multiplication of the feature vector, expressed in Equation (2) as:

(2) $$H^{\left(l+1\right)}=\sigma ({\tilde{D}}^{-1/2}\;\tilde{A}\;{\tilde{D}}^{-1/2}\;X\;\theta^{\left(l\right)})$$

where $\tilde{A}$ is the addition of the adjacency matrix *A* with its identity matrix $I_{N}$ to achieve self feature connection. $\tilde{D}$ and ${\tilde{D}}^{-1/2}$ represent the node degree matrix and its inverse for matrix scaling, respectively. *X* is the input representing the feature matrix, θ represents learnable parameters, *l* is the number of layers in the GCN, and σ is the nonlinear sigmoid function.

The inverse of the degree matrix, ${\tilde{D}}^{-1/2}$ , is multiplied on both sides of $\tilde{A}$ to get a weighted average of the nodes such that low degree and high degree nodes have an appropriate influence on their neighbors. As in our model, a two-layer GCN was stacked on each other to learn the complex spatial dependence of the graph network, and this can be expressed in Equation (2) as:

(3) $$f\left(X,A\right)=\sigma (\hat{A}\;\;ReLU\left(\hat{A}\;X\;W_{0}\right)\;W_{1})$$

where $\hat{A}={\tilde{D}}^{-1/2}\;\tilde{A}\;{\tilde{D}}^{-1/2}$ , $W_{0}$ is the weight matrix for the first GCN layer, $W_{1}$ is the weight matrix for the second GCN layer, and ReLU is the network’s activation function.

### 3.2. Gated Recurrent Unit (GRU)

In the time series data, we take advantage of the recurrent neural network’s memory cells to model the temporal dependence of traffic sequence forecasting. Having extracted the spatial features and dependence of the data using the GCN, the series is fed to a gated recurrent unit for sequence modeling while considering the previous time step in the prediction.

Given a sequence, the GRU uses the update gate and reset gate to create a hidden state of the current time step in conjunction with the previous hidden state, allowing it to maintain relevant information from a distant step without eroding over time. This helps the GRU to overcome the vanishing gradient problem of a standard recurrent neural network.

Given an input sequence at time *t*, the update gate, $u_{t}$ , determines how much information from the earlier time steps is rolled into the future, while the reset gate, $r_{t}$ , decides how much of the preceding information is forgotten. Both gates are calculated in Equations (4) and (5) as:

(4) $$u_{t}=\sigma \left(W_{u}*\left[X_{t},h_{t-1}\right]+b_{u}\right)\;$$

(5) $$r_{t}=\sigma \left(W_{r}*\left[X_{t},h_{t-1}\right]+b_{r}\right)$$

where $X_{t}$ is the input *X* at time *t*, $h_{t-1}$ is the hidden state from previous time step t−1 , *W* is the trainable weight, *b* is the trainable bias, and σ is the sigmoid activation function employed to squash the resulting output between 0 and 1. The two gates are then combined to obtain a new memory content, $c_{t}$ , and output state, $h_{t}$ , of time *t* as shown in Equations (6) and (7).

(6) $$c_{t}=tanh\left(W_{c}\;\left[X_{t},\left(r_{t}*h_{t-1}\right)\right]+b_{c}\right)\;$$

(7) $$h_{t}=u_{t}*h_{t-1}+\left(1-u_{t}\right)+c_{t}.$$

### 3.3. Attention Model

As evident by the outstanding results achieved in computer vision, natural language processing, and other areas of artificial intelligence, attention has presented dynamic ways of adaptively focusing on pertinent features of the input data. Extending the attention mechanism to capture spatiotemporal features, we applied multi-head attention to our GCN–GRU model to embed a spatial and temporal representation of the processed input sequence.

This ensures that the model sequence analysis focuses on the most significant features of traffic forecasts at each time step, based on the incorporation of relevant traffic properties such as individual road nodes in the road network and their interconnections to other road nodes.

First, the input data, *X*, is fed to the GCN to extract the spatial dependence and then to the GRU to model the temporal features, producing hidden states, $h_{i}\left(i=1,2,\cdots ,n\right)$ . To train and weigh the importance of the hidden states, the hidden states vector is fed into a two-layer single multi-head attention. The multi-head attention consists of query, key, and value heads, each consisting of a two-layer multilayer perceptron to obtain a scoring function adopted to compute the score of the output hidden state ( $H=h_{1},h_{2},\cdots ,h_{n})$ via a softmax function $\alpha_{ij}$ . Using a two-layer feed-forward network, $e_{ij}$ and the softmax $\alpha_{ij}$ are computed as shown in Equations (8) and (9), respectively.

(8) $$e_{ij}=w_{2}\left(w_{1}H+b_{1}\right)+b_{2}\;$$

(9) $$\alpha_{ij}=\frac{exp(e_{ij})}{\sum_{k=1}^{n}exp\left(e_{ik}\right)}$$

where *H* is the final hidden state output and *w* and *b* are learnable weights and bias for feed-forward network layers 1 and 2, respectively. The scores are multiplied by the hidden state vectors, *h*, and then summed to obtain the attention context vector $C_{t}$ .

(10) $$C_{t}=\sum_{j=1}^{n}\alpha_{ij}\;h_{j}$$

### 3.4. Loss Function

To estimate the network parameters, we utilize a loss function to optimize the trained parameters by reducing the error in each iteration. A modified mean absolute error (MAE), as suggested in [23], is defined in Equation (11).

(11) $$\ell =\left|\right|Y_{t}-{\hat{Y}}_{t}\left|\right|+\lambda L_{reg}$$

where $Y_{t}$ is the absolute real traffic speed values, ${\hat{Y}}_{t}$ represents the model’s predicted absolute values, λ is a learnable hyperparameter, and $L_{reg}$ is the $L_{2}$ regularization function which measures the loss regularization rate.

### 3.5. Overview

For a high-level intuition of the proposed model illustrated in Figure 2, MHSA–GCN is modeled for predicting traffic forecasts based on the graph convolutional network design, the recurrent neural network’s gated recurrent unit, and the multi-head attention mechanism, all combined to capture the complex topological structure of the road network effectively. The GCN is a compelling neural network architecture capable of producing useful feature representations of grid-like structures such as the road network. Thus, a GCN is used to extract road spatial relationships and connections, given adjacency and feature matrices. The aggregated representation of nodes is then fed to the gated recurrent unit for modeling the sequential time series features for temporal dependence. The attention mechanism is finally incorporated to ensure a particular focus is applied to the most significant features which cause the most considerable impact on the traffic forecast. As a supervised learning task, the model is trained iteratively, while the loss of the predicted values to the correct values is minimized via the update of learnable parameters through back propagation.

## 4. Experimental Analysis

### 4.1. Dataset

For evaluation, two real-world traffic forecast datasets were used in this work: the taxi trajectory dataset (SZ-taxi) and the loop detector dataset (Los-loop). Traffic midblock segments are not considered in this work, which relate to estimated traffic analyses at designated pedestrian intersections. The analysis included in this research investigates traffic prediction in flowing traffic.

The SZ-taxi dataset contains a taxi trajectory record of 156 major roads in the Luohu district of Shenzhen City, China. The data were collected from 1 to 31 January 2015, with each row representing a single road. About a thousand taxis participated in the data upload, which includes the vehicles’ latitude–longitude location and instantaneous speed measurements.

Framed as a graph convolution task, the model requires an adjacency matrix and a features matrix as inputs. Therefore, since there are 156 major roads, the adjacency matrix is a 156 × 156 matrix, with a value of 1 indicating connections between two roads and 0 indicating a lack of connection. Furthermore, the feature matrix is a 156 × 2976 matrix representing the change in traffic speed over time. The traffic speed was aggregated every 15 min, resulting in 2976 records over 31 days.

Secondly, the Los-loop data were recorded on 207 highway loop sensors from Los Angeles County from 1 to 7 March 2017 every 30 s. The data were then aggregated into 5-min intervals, so the adjacency matrix is a 207 × 207 matrix, while the feature matrix is a 207 × 2016 matrix.

### 4.2. Metrics

To assess the performance of the proposed MHSTA–GCN model, we evaluated the model on the Los-loop and SZ-taxi datasets. First, the error, which is the difference between the predicted values and the actual values, were computed and then the following metrics were examined and expressed. For example, in Table 1, the proposed MHSTA–GCN model was evaluated on the SZ-taxi and Los-loop test data for traffic prediction ranging from 15 min to 60 min.

1. Mean absolute error (MAE): The MAE is also known as the $L_{1}$ -norm loss and is computed as the mean sum of the absolute error, which is the absolute difference between the absolute values and the actual values. The MAE is non-negative, and it is more suitable for penalizing smaller errors. The best MAE score is 0.0, and it is computed as shown in Equation (12).
(12) $$\mathrm{MAE}=\frac{\sum_{i=1}^{n}\left|y_{t}-{\hat{y}}_{t}\right|}{n}$$
2. Root mean square error (RMSE): The RMSE is the square root of the squared error differences, and it measures the standard deviation of the predicted errors or how spread out the errors are. This details how concentrated the predicted data are along the line of best fit and is computed as shown in Equation (13).
(13) $$\mathrm{RMSE}=\sqrt[{\frac{1}{2}}]{\frac{1}{n}\sum_{t=1}^{n}{\left(y_{t}-{\hat{y}}_{t}\right)}^{2}}$$
3. The $R^{2}$ score or the coefficient of determination expresses the distribution of the variance of actual labels that the independent variables in the model have interpreted. The best $R^{2}$ score is 1, and it measures the model’s capability to predict new data correctly. It is computed as shown in Equation (14).
(14) $$R^{2}=1-\frac{\sum_{t=1}{\left(y_{t}-{\hat{y}}_{t}\right)}^{2}}{\sum_{t=1}{\left(y_{t}-{\bar{y}}_{t}\right)}^{2}}$$
4. Explained variation (VAR): Explained variation measures the proportion of model variation influenced by actual factors in the data rather than the error variance. It is computed as shown in Equation (15).
(15) $$\mathrm{VAR}=1-\frac{Var(y_{t}-{\hat{y}}_{t})}{Var(y)}$$
5. Accuracy: Accuracy describes how close the model prediction is to the actual values, whereby a perfect accuracy would result in a score of 1. It is defined as shown in Equation (16).
(16) $$\mathrm{Accuracy}=1-\frac{||y-\hat{y}{\left|\right|}_{F}}{{\left||y|\right|}_{F}}$$
  where *y* is the actual traffic value, $\hat{y}$ is the predicted value, $\bar{y}$ is the average of *y*, and ${\left||.|\right|}_{F}$ represents the Frobenius norm.

### 4.3. Training Details

The MHSA–GCN model consists of a graph convolutional network, a gated recurrent unit, and an attention mechanism to give traffic forecasts based on traffic speed. The model was trained for 5000 epochs, with a training batch size of 32, a learning rate of 0.001, and an Adam optimizer to minimize the computed loss.

For the SZ-taxi dataset, the model’s hidden unit was set to 100, while the hidden unit for the Los-loop dataset was set to 64. These specific values were used as the most optimal values for obtaining a good result without fear of underfitting or overfitting. This is achieved using machine learning tuner mechanisms and regularizers such as dropout.

To verify the performance efficiency of the model, the complete dataset was partitioned into a train–test split, whereby 80% of the data were used for model training while the remaining 20% were used for model testing. Furthermore, the model prediction for both datasets was forecasted for the next 15, 30, 45, and 60 min, respectively.

For the two-layer multi-head attention model, since the recurrent network’s hidden unit for the SZ-taxi dataset was 100, the attention model’s first layer was set to 100 neurons, while the second layer was set to 156—the number of major roads in the data. Similarly, for the Los-loop dataset, the first and second layers of the attention neurons were set to the number of recurrent hidden units and numbers of sensors in the data, respectively.

Since the model is trained as an end-to-end network, the model loss is computed with each iteration and the weights are updated to minimize the loss, therefore refining the model’s ability to make accurate predictions.

### 4.4. Baselines

The following baselines were compared to the MHSA–GCN model to investigate the effects of the model parameters and hyperparameters and how they translate to prediction accuracy.

1. History average model (HA) [48]: this technique computes the prediction by averaging the total amount of available historical data by classifying them into periods.
2. Graph convolutional network model (GCN) [49]: the GCN model uses the feature and adjacency matrix attributes to model the spatial features of the traffic data by treating the roads as nodes alongside their connectivity.
3. Gated recurrent unit model (GRU) [34]: to handle the temporal complexity of the urban roads, a recurrent model with a gated flow of states is implemented to follow the sequential time analysis.

### 4.5. Experimental Results

As shown in Table 2, the MHSA–GCN model presents the best prediction performance of the highlighted models for almost all of the predicted periods of 15, 30, 45, and 60 min. This shows the effectiveness and efficiency of the MHSA–GCN model in modeling the complex spatiotemporal topological structure of the road and traffic data, indicating how important it is to combine both the spatial and temporal feature representations to establish a learning feature.

For instance, compared to the HA model for the 15 min forecast on the SZ-taxi and Los-loop datasets, the MHSA–GCN model exhibited a reduction of 4.28% and 7.50% in the MAE error, respectively. Furthermore, for the RMSE error for the 60 min prediction, the MHSA–GCN model exhibits a reduction of 7.62% and 5.73%, respectively, compared to the HA model. Since the HA model only averages the historical traffic data to forecast traffic, it cannot learn the seasonality and trends of the data, which constitute a significant component of time series data.

Furthermore, the MHSA–GCN model exhibits increased accuracy and explained variation (VAR) compared to the other models. For both the SZ-taxi and Los-loop datasets, the MHSA–GCN model exhibits an increase of 17% and 5.5% compared to the GCN, as well as a 1.67% and 0.81% increase compared to the GRU model for the 15 min prediction. For the 60 min prediction, the MHSA–GCN model also exhibits an increase of 16.7% and 4.43% compared to the GCN, as well as a 1.06% and 1.43% increase compared to the GRU model.

The increased accuracy values compared to the GCN and the GRU indicate the advantage of combining both the spatial and temporal feature representations of the data rather than exploring just one of them, such as in the GCN (spatial features only) and in the GRU (temporal features only). The explained variance, which suggests how much of the model variation is influenced by fundamental factors in the data rather than the error variance, reinforces this. The MHSA–GCN model also displayed superior outcomes in the explained variance values compared to the other models. This is because the MHSA–GCN model can learn the mapping of the variations in the actual data based on seasonality, trends, and their resulting peaks and troughs.

### 4.6. Ablation Study

To demonstrate the dynamic efficiency of the appended multi-head spatiotemporal attention on the graph convolutional network, we compared the ablation experiment of MHSA–GCN with the following models.

1. Temporal graph convolutional network (T-GCN) [34]: this technique also combines a recurrent network with a graph convolutional network to manage the complex spatiotemporal topological structure; however, any attention model is used.
2. Attention temporal graph convolutional network (A3T-GCN) [38]: the A3T-GCN model explores the impact of a different attention mechanism (soft attention model) on traffic forecasts.

Without an attention mechanism, the T-GCN model forecast short-term and long-term traffic forecasts better than the HA, GCN, and GRU models. However, in comparison to the MHSA–GCN model, the results are less accurate. This proves the capability of the attention mechanism to increase the robustness of the model and the ability to capture global variation effectively.

For example, the MHSA–GCN model exhibits a 1.82% and 5.29% reduction in the MAE error on the SZ-taxi and Los-loop dataset (15 min prediction) compared to the T-GCN model. Furthermore, for the 60 min prediction, the MHSA–GCN model exhibits a reduction of 1.89% and 8.32%, respectively, in the MAE error compared to the T-GCN model. Similarly, MHSA–GCN also has a better RMSE error, showing that the model converges better to find the global minima and updates the weights to achieve a lower loss and therefore better accuracy.

Considering the 15 min prediction, 3.18% and 4.78% reductions in the RMSE error compared to the T-GCN model are recorded by the MHSA–GCN model on the SZ-taxi and Losloop datasets, while 0.93% and 3.46% reductions are recorded for the 60 min prediction. This confirms that MHSA–GCN has both a better short-term and long-term prediction capability than the T-GCN model, showing the superiority of the attention mechanism. This is because the attention model helps the network focus on features that are the most significant to the traffic forecast, and it also shows superiority in capturing global variation.

Although the A3T-GCN model incorporates an attention mechanism, the MHSA–GCN achieves better results compared to this model as well; this is because of the differences in the dynamic capability of the different attention mechanisms to capture global variation. The A3T-GCN model implements soft attention, while the MHSA–GCN model implements multi-head attention, consisting of three attention heads or modules that run several computations in parallel, dynamically attending to sections of a sequence differently. This results in an advantageous effect of mapping varying features such as longer-term dependencies and shorter-term dependencies.

Considering the 15 min prediction, 0.81% and 3.95% reductions in the MAE error compared to the A3T-GCN model are recorded by the MHSA–GCN model on the SZ-taxi and Losloop datasets, respectively, while 0.11% and 0.36% reductions are recorded for the 60 min prediction. In a similar manner, MHSA–GCN also has a better RMSE error, with 2.49% and 4.11% error reductions for the SZ-taxi and Los-loop datasets, respectively, for the 15 min prediction, as well as 0.07% and 1.16% error reductions for the 60 min prediction.

While the differences between the A3T-GCN and MHSAGCN model in the smaller prediction range, such as 15 min, are very small, the differences between both models in more extended predictions such as 60 min are quite significant. This shows that although the A3T-GCN model can learn shorter-term dependencies, the MHSA–GCN model displays superiority, especially with longer-term dependencies.

### 4.7. Visualization Analysis

To extensively understand the forecasting capability of the MHSA–GCN model, we visualized the model’s predictions in comparison to the actual values of traffic for specific periods. This was done for both the SZ-taxi and Los-loop datasets for the 15, 30, 45, and 60 min time series prediction.

**Figure 3:**
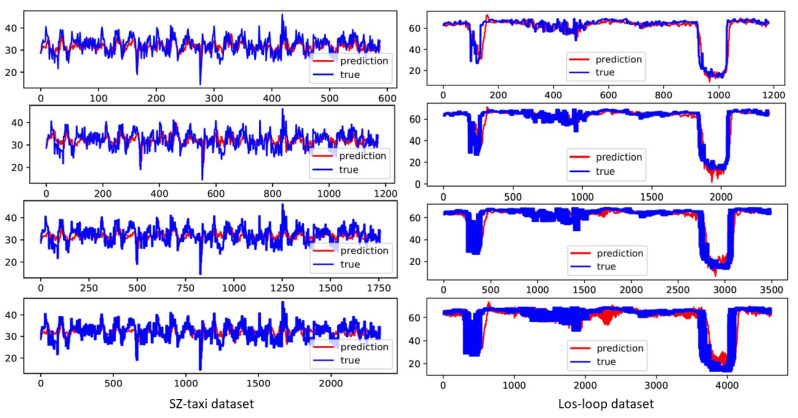
MHSA–GCN traffic prediction display compared to the true values for 15, 30, 45, and 60 min from top to bottom on both SZ-taxi and Los-loop datasets.

**Figure 4:**
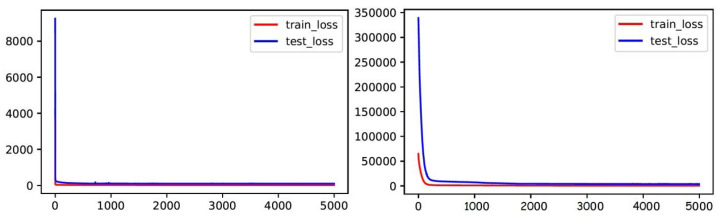
Train and test loss graph for the 15 minutes prediction for the SZ-taxi (**right**) and Los-loop (**left**) datasets.

As shown in Figure 3 and Figure 4, the visualization shows that the predicted traffic speed depicts comparable trends and flow to the actual traffic values for the predicted periods. This further validates the capability of the MHSA–GCN model to map varying features of shorter-term dependencies and longer-term dependencies and to capture global variation in traffic flow efficiently. Furthermore, during training, the model is able to continuously converge to the global minimum to achieve a better prediction accuracy, as depicted in Figure 4.

## 5. Conclusions

This paper proposed a novel automatic traffic prediction model named multi-head spatiotemporal attention graph convolutional network (MHSTA–GCN), which combines a graph convolutional network (GCN), a gated recurrent unit (GRU), and a multi-head attention module to learn feature representation of road traffic speed as nodes in a connected network. Given the interconnected nodes and historical traffic speed data, the GCN was used to learn the fundamental attributes of the nodes by capturing the complex spatial topological structure of the graph. Furthermore, the GRU model was introduced to understand the dynamic temporal dependence of the node attributes. Finally, the multi-head attention mechanism was employed to give more consideration to the significant features that contribute more to the traffic state at a given time. Thus, the dynamic variation in the time steps is sequentially and efficiently analyzed to capture and incorporate the global variation over time.

Combining these three modules proved to be an effective and robust model for capturing the essential spatio-temporal sequential features for traffic prediction, as evaluated on the SZ-taxi and Los-loop datasets. With 91.8% and 89.9% accuracy on the Los-loop data for 15- and 30-min prediction, and an R2 score of 0.85 on the SZ-taxi dataset for the 15- and 30-min prediction, the MHSTA–GCN model performance demonstrates state-of-the-art traffic forecasting and superiority compared to other traffic prediction models.

As with most large machine learning models, the process of iteratively finetuning the model’s hyperparameters can be quite daunting and often affects the model’s ability to reach the global minimum. Therefore, in the future, we will extensively investigate the possibility of using an attention network to dynamically incorporate external factors such as weather, population, and geographical features for traffic prediction with the use of a graph convolutional network. Furthermore, the advantage of midblock segments, traffic peaks and troughs, and road lane availability will be explored.

## Figures and Tables

**Table 1:** MHSA–GCN traffic prediction evaluation on the SZ-taxi and Los-loop datasets.

Time (min)	Metric	SZ-Taxi	Los-Loop
	$R^{2}$	0.8553	0.8659
	Var	0.8553	0.8659
15	MAE	2.6624	3.0126
	RMSE	3.8017	4.8812
	Accuracy	0.7372	0.9183
	$R^{2}$	0.8499	0.8153
	Var	0.8499	0.8153
30	MAE	2.7101	3.4800
	RMSE	3.8801	5.6744
	Accuracy	0.7360	0.8992
	$R^{2}$	0.8471	0.7694
	Var	0.8473	0.7694
45	MAE	2.7221	4.1098
	RMSE	3.9014	6.6127
	Accuracy	0.7301	0.8875
	$R^{2}$	0.8466	0.7521
	Var	0.8466	0.7553
60	MAE	2.7361	4.2190
	RMSE	3.9678	7.0165
	Accuracy	0.7274	0.8812

**Table 2:** MHSA–GCN traffic prediction comparison with other models on SZ-taxi and Los-loop datasets. Higher accuracies are in bold.

Metric	Time	SZ-Taxi						Los-Loop					
		HA [48]	GCN [49]	GRU [34]	T-GCN [34]	A3T-GCN [38]	MHSA–GCN	HA [48]	GCN [49]	GRU [34]	T-GCN [38]	A3T-GCN [27]	MHSA–GCN
$R^{2}$	15 min	0.8307	0.6654	0.8329	0.8541	0.8512	**0.8553**	0.7121	0.6843	0.8576	0.8634	0.8653	**0.8659**
	30 min	0.8307	0.6616	0.8249	0.8456	0.8493	**0.8499**	0.7121	0.6402	0.7957	0.8098	0.8137	**0.8153**
	45 min	0.8307	0.6589	0.8198	0.8441	**0.8474**	0.8471	0.7121	0.5999	0.7446	0.7679	0.7694	**0.7694**
	60 min	0.8307	0.6564	0.8266	0.8422	0.8454	**0.8466**	0.7121	0.5583	0.6980	0.7283	0.7407	**0.7521**
Var	15 min	0.8307	0.6655	0.8329	0.8541	0.8512	**0.8553**	0.7121	0.6844	0.8577	0.8634	0.8653	**0.8659**
	30 min	0.8307	0.6617	0.8250	0.8457	0.8493	**0.8499**	0.7121	0.6404	0.7958	0.8100	0.8137	**0.8153**
	45 min	0.8307	0.6590	0.8199	0.8441	**0.8474**	0.8473	0.7121	0.6001	0.7451	0.7684	**0.7705**	0.7694
	60 min	0.8307	0.6564	0.8267	0.8423	0.8454	**0.8466**	0.7121	0.5593	0.6984	0.7290	0.7415	**0.7553**
MAE	15 min	2.7815	4.2367	2.5955	2.7117	2.6840	**2.6624**	4.0145	5.3525	3.0602	3.1802	3.1365	**3.0126**
	30 min	2.7815	4.2647	2.6906	2.7410	**2.7038**	2.7101	4.0145	5.6118	3.6505	3.7466	3.6610	**3.4800**
	45 min	2.7815	4.2844	2.7743	2.7612	2.7261	**2.7221**	4.0145	5.9534	4.0915	4.1158	4.1712	**4.1098**
	60 min	2.7815	4.3034	2.7712	2.7889	2.7391	**2.7361**	4.0145	6.2892	4.5186	4.6021	4.2343	**4.2190**
RMSE	15 min	4.2951	5.6596	3.9994	3.9265	3.8989	**3.8017**	7.4427	7.7922	5.2182	5.1264	5.0904	**4.8812**
	30 min	4.2951	5.6918	4.0942	3.9663	3.9228	**3.8801**	7.4427	8.3353	6.2802	6.0598	5.9974	**5.6744**
	45 min	4.2951	5.7142	4.1534	3.9859	3.9461	**3.9014**	7.4427	8.8036	7.0343	6.7065	6.6840	**6.6127**
	60 min	4.2951	5.7361	4.0747	4.0048	3.9707	**3.9678**	7.4427	9.2657	7.6621	7.2677	7.0990	**7.0165**
Accuracy	15 min	0.7008	0.6107	0.7249	0.7299	0.7318	**0.7372**	0.8733	0.8673	0.9109	0.9127	0.9133	**0.9183**
	30 min	0.7008	0.6085	0.7184	0.7272	0.7302	**0.7360**	0.8733	0.8581	0.8931	0.8968	0.8979	**0.8992**
	45 min	0.7008	0.6069	0.7143	0.7258	0.7286	**0.7301**	0.8733	0.8500	0.8801	0.8857	0.8861	**0.8875**
	60 min	0.7008	0.6054	0.7197	0.7243	0.7269	**0.7274**	0.8733	0.8421	0.8694	0.8762	0.8790	**0.8812**

## Data Availability

Not applicable.
