# Correction: Oluwasanmi et al. Multi-Head Spatiotemporal Attention Graph Convolutional Network for Traffic Prediction. *Sensors* 2023, *23*, 3836

**DOI:** 10.3390/s24248214

**Published:** 2024-12-23

**Authors:** Ariyo Oluwasanmi, Muhammad Umar Aftab, Zhiguang Qin, Muhammad Shahzad Sarfraz, Yang Yu, Hafiz Tayyab Rauf

**Affiliations:** 1 School of Information and Software Engineering, University of Electronic Science and Technology of China, Chengdu 610054, China; 2 Department of Computer Science, National University of Computer and Emerging Sciences, Islamabad, Chiniot-Faisalabad Campus, Chiniot 35400, Pakistan; 3 Centre for Infrastructure Engineering and Safey, School of Civil and Environmental Engineering, The University of New South Wales, Sydney, NSW 2052, Australia; 4 Independent Researcher, Bradford BD8 0HS, UK

## Affiliation Correction

In the original publication [1], the sixth author’s Affiliation 4 was wrong. The correct Affiliation 4 appears below:

Independent Researcher, Bradford BD8 0HS, UK

The authors state that the scientific conclusions are unaffected. This correction was approved by the Academic Editor. The original publication has also been updated.

## References

[1] Oluwasanmi A., Aftab M.U., Qin Z., Sarfraz M.S., Yu Y., Rauf H.T. (2023). Multi-Head Spatiotemporal Attention Graph Convolutional Network for Traffic Prediction. Sensors.

